# Node-degree aware edge sampling mitigates inflated classification performance in biomedical random walk-based graph representation learning

**DOI:** 10.1093/bioadv/vbae036

**Published:** 2024-03-04

**Authors:** Luca Cappelletti, Lauren Rekerle, Tommaso Fontana, Peter Hansen, Elena Casiraghi, Vida Ravanmehr, Christopher J Mungall, Jeremy J Yang, Leonard Spranger, Guy Karlebach, J Harry Caufield, Leigh Carmody, Ben Coleman, Tudor I Oprea, Justin Reese, Giorgio Valentini, Peter N Robinson

**Affiliations:** AnacletoLab, Dipartimento di Informatica, Università degli Studi di Milano, Milano 20133, Italy; The Jackson Laboratory for Genomic Medicine, CT 06032, United States; AnacletoLab, Dipartimento di Informatica, Università degli Studi di Milano, Milano 20133, Italy; The Jackson Laboratory for Genomic Medicine, CT 06032, United States; AnacletoLab, Dipartimento di Informatica, Università degli Studi di Milano, Milano 20133, Italy; Division of Environmental Genomics and Systems Biology, Lawrence Berkeley National Laboratory, Berkeley, CA 94710, United States; The Jackson Laboratory for Genomic Medicine, CT 06032, United States; Division of Environmental Genomics and Systems Biology, Lawrence Berkeley National Laboratory, Berkeley, CA 94710, United States; Department of Internal Medicine and UNM Comprehensive Cancer Center, UNM School of Medicine, Albuquerque, NM 87102, United States; Institute of Bioinformatics, Freie Universität Berlin, Berlin, 14195, Germany; The Jackson Laboratory for Genomic Medicine, CT 06032, United States; Division of Environmental Genomics and Systems Biology, Lawrence Berkeley National Laboratory, Berkeley, CA 94710, United States; The Jackson Laboratory for Genomic Medicine, CT 06032, United States; The Jackson Laboratory for Genomic Medicine, CT 06032, United States; Institute for Systems Genomics, University of Connecticut, Farmington, CT 06032, United States; Department of Internal Medicine and UNM Comprehensive Cancer Center, UNM School of Medicine, Albuquerque, NM 87102, United States; Division of Environmental Genomics and Systems Biology, Lawrence Berkeley National Laboratory, Berkeley, CA 94710, United States; AnacletoLab, Dipartimento di Informatica, Università degli Studi di Milano, Milano 20133, Italy; ELLIS—European Laboratory for Learning and Intelligent Systems; The Jackson Laboratory for Genomic Medicine, CT 06032, United States; Institute for Systems Genomics, University of Connecticut, Farmington, CT 06032, United States; ELLIS—European Laboratory for Learning and Intelligent Systems; Berlin Institute of Health, Charité – Universitätsmedizin Berlin, Berlin, 10117, Germany

## Abstract

**Motivation:**

Graph representation learning is a family of related approaches that learn low-dimensional vector representations of nodes and other graph elements called embeddings. Embeddings approximate characteristics of the graph and can be used for a variety of machine-learning tasks such as novel edge prediction. For many biomedical applications, partial knowledge exists about positive edges that represent relationships between pairs of entities, but little to no knowledge is available about negative edges that represent the explicit lack of a relationship between two nodes. For this reason, classification procedures are forced to assume that the vast majority of unlabeled edges are negative. Existing approaches to sampling negative edges for training and evaluating classifiers do so by uniformly sampling pairs of nodes.

**Results:**

We show here that this sampling strategy typically leads to sets of positive and negative examples with imbalanced node degree distributions. Using representative heterogeneous biomedical knowledge graph and random walk-based graph machine learning, we show that this strategy substantially impacts classification performance. If users of graph machine-learning models apply the models to prioritize examples that are drawn from approximately the same distribution as the positive examples are, then performance of models as estimated in the validation phase may be artificially inflated. We present a degree-aware node sampling approach that mitigates this effect and is simple to implement.

**Availability and implementation:**

Our code and data are publicly available at https://github.com/monarch-initiative/negativeExampleSelection.

## Introduction

Many problems in biology and medicine stand to benefit from machine learning (ML) approaches (Rajkomar *et al.* 2019). Biomedical data are often composed of entities from multiple different classes that are interconnected by different types of relation. Therefore, biological data are often represented computationally as knowledge graphs (KG), semantic networks that encode entities as nodes and relations between entities as edges. Typical ML tasks that leverage KGs involve node (entity) classification and prediction of novel relations between entities (edge prediction) (Nickel *et al.* 2016, Li *et al.* 2022). Graph machine learning methods have been applied to numerous biomedical classification tasks including protein function prediction, protein–protein interaction prediction and *in silico* drug discovery Muzio *et al.* (2021). Despite the great promise of ML in medicine, to date very few ML algorithms have contributed meaningfully to clinical care Deo (2015). One reason for this might be that published models not infrequently display methodological flaws or underlying biases (Zech *et al.* 2018, Biderman and Scheirer 2020; Wynants *et al.* 2020, Roberts *et al.* 2021). It is therefore essential to understand and ideally to mitigate sources of bias and error in ML in order to develop robust and accurate algorithms.

It was shown in 2011 that topological imbalances in biomedical KGs can result in densely-connected entities (i.e. high-degree nodes) being highly ranked no matter the context, suggesting that embedding models may be more influenced by node degree than by any biological information encoded within the relations (Gillis and Pavlidis 2011, Bonner *et al.* 2022). Here, we show that the method by which negative edges are sampled for evaluation of results in the validation phase can contribute to this node-degree bias. We present an approach to mitigating the effect by node-degree aware sampling. We demonstrate our approach using two heterogeneous KGs.

### Definitions

A graph G=(V,E) consists of nodes (a.k.a. vertices) v∈V and edges (a.k.a. links or relations) $e_{u,v}^{r}\in E$ connecting nodes *u* and *v* via a relationship of type *r*.

A graph with a single node type and a single edge type is called homogeneous. For example, the protein-protein interaction graph described below is homogeneous because it has one type of node (a gene symbol that represents the proteins encoded by the gene) and one type of edge (an interaction between a pair of proteins). Graphs with two or more types of node, two or more types of edge, or both are called heterogeneous. For instance, the synthetic lethality graph described below is heterogeneous because it contains two types of edges, one for protein-protein interactions and another for synthetic lethality interactions.

A knowledge graph is a graph that uses nodes to represent real-world entities and edges to represent the relations between these entities.

A random walk is defined as an iterative walker’s transition from its current node to a randomly selected neighbor starting at a given source node, *s*. In the experiments described here, we simulate random walks of a fixed path length l=128.

With a first-order random walk, if the walker is at node *n* at step *i*, the next random step is chosen based on information solely from the immediate neighbors of node *n*. With a second-order random walk, the next random step is chosen based on information from the previous random walk step and the immediate neighbors of node *n*.

Graph representation learning (GRL) is a form of graph machine learning that applies various strategies to convert nodes, edges, or graphs into low-dimensional vectors called “embeddings” that preserve graph structural information and properties (Cai *et al.* 2018). Graph embeddings can be used to address downstream prediction tasks (Xu 2021). In this work, we focus on random-walk based GRL methods that optimize node embeddings such that nodes have similar embeddings if they tend to co-occur on short random walks over the graph (Li *et al.* 2022, Hamilton *et al.* 2017).

Shallow embedding methods generate a vector representation for every node *u* that preserves the input graph structure information. The methods are called shallow to distinguish them from graph neural networks that can generate representations for any graph element by capturing both network structure and node attributes and metadata using deep learning techniques (Li *et al.* 2022). Numerous approaches have been developed to generate embeddings that reflect different aspects of graph structure (Perozzi *et al.* 2014, Mikolov *et al.* 2013b, Pennington *et al.* 2014, Tang *et al.* 2015, Grover and Leskovec 2016).

The Matthews correlation coefficient (MCC) is calculated based on the counts of true-positive (TP), true-negative (TN), false-positive (FP), and false-negative (FN) classifications as follows:

$$\begin{array}{c} \text{MCC}=\frac{\text{TP}\cdot \text{TN}-\text{FP}\cdot \text{FN}}{\sqrt{\left(\text{TP}+\text{FP})\cdot (\text{TP}+\text{FN})\cdot (\text{TN}+\text{FP})\cdot (\text{TN}+\text{FN}\right)}} \end{array}$$

The MCC generates a high score only if the binary predictor was able to correctly predict the majority of positive data instances and the majority of negative data instances, with its values ranging from –1 for perfect misclassification to +1 for perfect classification. MCC = 0 is the expected value for random classification (Chicco and Jurman 2020).

### Protein–protein associations

The STRING database is a comprehensive relational database of protein-protein associations (Szklarczyk *et al.* 2021). STRING (version 11.0) data for *Homo sapiens* was used corresponding to 9606.protein.links.v11.5.txt.gz. Associations were filtered to retain only those with a score of at least 700, and duplicate edges between node pairs were removed.

### Synthetic lethal interaction data

We first analyzed data available in the supplementary material provided with ISLE (Lee *et al.* 2018) and files available from the SynLethDB resource (Guo *et al.* 2016) by comparing the curation with the original publications. To create the synthetic lethal interaction database (SLDB) resource, we manually reviewed publications cited in these resources and additional publications. The curated SLIs are available at https://github.com/monarch-initiative/syntheticLethalityNetwork. The tab-separated file (TSV) includes information about each pair of genes, the perturbations used for each gene, the assays used to measure synthetic lethality, a Cellosaurus id (Bairoch 2018) (if applicable), and the PubMed identifier. To add additional information to SLDB, we integrated it with the STRING protein-protein interaction network by using the nodes (genes) in SLDB to also represent the proteins in STRING that the genes themselves encode.

For the experiments described in this work, we imported the SLDB resource directly using a utility function of GRAPE (Cappelletti *et al.* 2023). The SLDB graph was integrated with the STRING PPA graph. A Python script that implements the analysis is available in the project GitHub repository (runSli.py).

### KG-IDG

The KG-IDG knowledge graph represents data from the NIH Common Fund’s Illuminating the Druggable Genome (IDG) Consortium. The IDG aims to integrate current knowledge of proteins in order to study the function of specific understudied drug targets in three main druggable protein families: G-protein coupled receptors, ion channels and protein kinases. KG-IDG is intended to represent relationships between drugs, their protein targets, and disease. KG-IDG unifies structured data from 14 different sources concerning drugs, proteins, and diseases (Caufield *et al.* 2023b). For experiments in which we trained edge prediction models on KG-IDG, we used edges between biolink: ChemicalSubstance, biolink: ChemicalEntity, biolink: Drug nodes and biolink: Protein nodes for training and to test the performance of the edge prediction model.

### Shallow graph representation learning

The shallow graph representation learning experiments described here were performed using the GRAPE library for fast and scalable Graph Processing and Embedding, version 0.1.28. GRAPE provides a comprehensive library of Graph Representation Learning and inference models implemented in Rust with a Python interface (Cappelletti *et al.* 2023). Seven node embedding methods were used including four random-walk methods and three matrix factorization methods.

The random-walk methods combine methods for generating random walks with methods for sampling node contexts. The two random walk sampling mechanisms are DeepWalk (Perozzi *et al.* 2014), which samples first-order random walks, and Walklets (Perozzi *et al.* 2017), which samples first-order random walks and, for a given central node *v*, at each *i*-th sampling iteration, skips *i* nodes around the central node *v*. We used these training samples obtained with both DeepWalk and Walklets to train two different embedding models. The CBOW (Mikolov *et al.* 2013a) model, trains a shallow neural network to predict the central node of a random walk window given the remainder contextual nodes. The second model is SkipGram (Mikolov *et al.* 2013a), which analogously to CBOW trains a shallow neural network to predict the contextual nodes given the central node. In all of these models, the node embedding matrix is the (trained) weight matrix of the first hidden layer.

The matrix factorization methods included Large-scale Information Network Embedding (LINE) and High-Order Proximity preserved Embedding (HOPE). First and second-order LINE (Tang *et al.* 2015) trains a neural network with either one layer (first-order) or two layers (second-order) to predict whether a given tuple of nodes defines an existing edge. HOPE starts by computing a node-proximity matrix, where the proximity between two nodes may be defined in different ways, in our case by using the number of common neighbors. Then HOPE computes the singular vectors corresponding to the *k* most significant singular values of the proximity matrix and uses the left and right product of the singular values with the singular vectors as the embeddings of the source and destination nodes (Ou *et al.* 2016).

Each of the seven algorithms was used with GRAPE default parameters. Details are provided in Table 1.

**Table 1.** Parameters for the learning models used in this project.

Model	Epochs	Learning rate	Walk length	Window size	Max neighbors
DeepWalk CBOW	30	0.010	128	5	100
DeepWalk SkipGram	30	0.010	128	5	100
Walklets CBOW	30	0.010	128	4	100
Walklets SkipGram	30	0.010	128	4	100
First-order LINE	100	0.050	n/a	n/a	n/a
Second-order LINE	100	0.050	n/a	n/a	n/a
HOPE	n/a	n/a	n/a	n/a	n/a

Each of the models shown here was run using uniform and node-based sampling of negative examples. In addition to the parameters shown in the table, some parameters are only relevant to a subset of models: learning rate decay was set to 0.9. Avoid false negatives was set to false for First-order LINE and Second-order LINE. The number of negative samples was set to 10 for DeepWalk CBOW, DeepWalk SkipGram, Walklets CBOW, and Walklets SkipGram. Iterations were set to 100 for all models except first-order and second-order LINE.

#### Edge prediction

Edge embeddings were formed from the embeddings of the corresponding pair of nodes (u,v) by the binary Hadamard (⊡) operator, defined as the elementwise product of both vectors, i.e.

$$\begin{array}{c} {\left[f(u)⊡f(v)\right]}_{i}=f_{i}(u)\times f_{i}(v) \end{array}$$

Edge prediction was performed by a Perceptron model. Positive edges for training and evaluation were derived from the SLDB KG, and negative edges were obtained as will be described in the Results.

## Results

**Figure 1.**
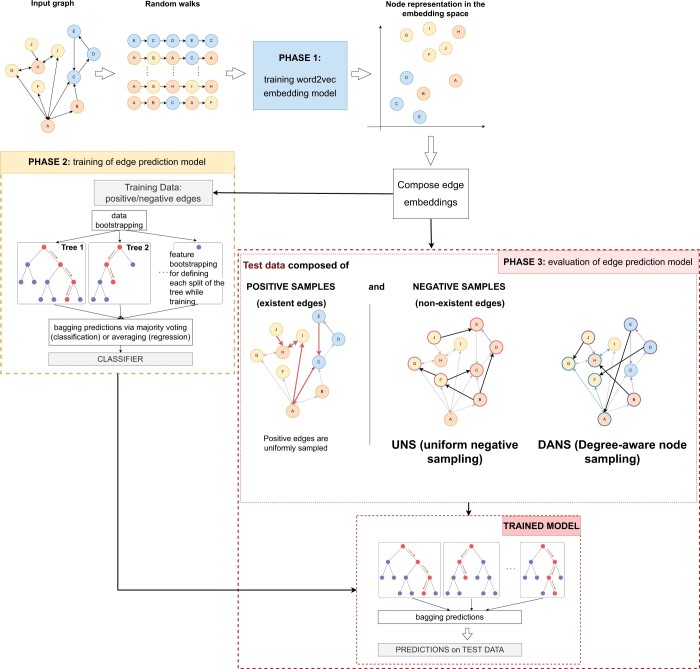
Graph representational learning and link prediction. There are three phases in which negative examples are sampled. In the first, embeddings are generated from the input graph using a variety of methods that sample the graph by random walks or related procedures. Edge embeddings are then generated from the node embeddings. In the second phase, a classifier (such as a random forest) is created using the positive and negative labels from a training set. In the third phase, the resulting classifier is *evaluated* on the basis of its performance on held-out data. In typical bioinformatics applications, we have knowledge about a subset of positive examples, and assume all unlabeled examples are negative. In the current work, we explore two strategies (UNS and DANS) for sampling from unlabeled examples to obtain negative examples for evaluation.

Here, we investigate the influence of negative-edge sampling in link prediction by graph ML. The relevant algorithms have three main stages, each of which uses a different type of negative sampling (Fig. 1). As we will explain below, the sampling strategy that has been traditionally used in the third stage can artificially inflate the measured classification performance for new data that have a distribution similar to that of the positive training set. In this work, we present a degree-aware node sampling approach for sampling negative edge examples that mitigates this effect. Before we discuss our approach, we will present a brief explanation of the three phases.

In the first (embedding) phase, embeddings are generated from the input graph. The embedding procedure is a generalization of the word embedding procedure of the word2vec algorithm (Mikolov *et al.* 2013b), whereby each random walk is like a sentence and the nodes of the graph are like words. Much previous work has been invested in understanding the effect of negative sampling in the first phase (training the embedding model). The computational objective of the skipgram model is to maximize the mean log-probability of context words that occur in a window surrounding the input word. For instance, the node2vec algorithm scans over series of nodes encountered in random walks and attempts to predict nearby nodes (i.e. inside some context window) on the basis of a Skip-gram objective function.

However, the per-node partition function is expensive to compute for large networks since it involves every node of the graph, and so node2vec approximates it using negative sampling, whereby *k* negative nodes are sampled for each positive node according to the unigram distribution U (w) raised to the 3/4rd power (Mikolov *et al.* 2013b, Grover and Leskovec 2016, Ahrabian *et al.* 2020; Yang *et al.* 2020; Wang *et al.* 2023). A number of methods have been developed to improve the selection of negative examples for creating embeddings (Zhang and Zweigenbaum 2018, Armandpour *et al.* 2019) but no single method performs best for all datasets (Caselles-Dupré *et al.* 2018, Yang *et al.* 2020). The first phase concludes with the generation of edge embeddings from the node embeddings, which can be performed with several methods (Grover and Leskovec 2016).

The method that we present in this work applies only to the second and third phases and is independent of this kind of negative sampling chosen for the first phase.

In the second (classification) phase, positive and labeled edge examples are extracted from the KG for training an edge classifier. For example, to classify protein-protein associations, positive edges can be obtained from the STRING knowledge base, and for the synthetic lethality graph, positive synthetic lethality interactions can be curated from the literature. In general, only a small proportion of all potential edges are labeled positive. For instance, the STRING graph has 16 812 nodes, corresponding to (16 812×16 811/2) potential edges between pairs of nodes, or roughly 141 million edges. Only 252 953 edges, or 0.18%, are labeled positive. To train machine-learning classifiers, it is recommended to use numbers of positive and negative examples that do not greatly differ. STRING does not include information about pairs of proteins that do not undergo interactions. For this reason, negative examples are sampled at random from the unlabeled set.

Many classification algorithms are suitable for the second phase. Figure 1 shows a Random Forest classifier. For the experiments shown here, we used a single layer neural network (i.e. Perceptron) for edge classification.

In the third (evaluation) phase, the performance of the classifier is evaluated. Similar to the second phase, a relatively balanced number of positive and negative examples are chosen for the evaluation.

The current work explores the consequences of two strategies for choosing negative examples in the third (evaluation) phase.

### Input knowledge graph

**Figure 2.**
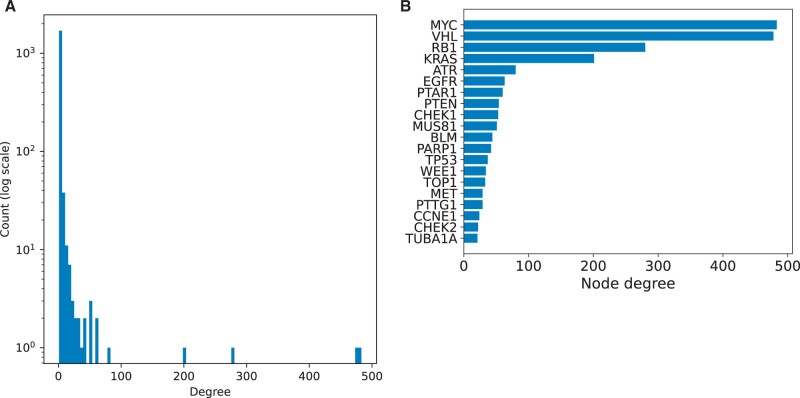
Topology of the SLDB graph. (A) Degree distribution of the synthetic lethality interaction edges in the SLDB graph. (B) The 20 highest-degree nodes with synthetic lethality interactions of the SLDB network. The distribution of node degrees among the 20 most densely connected nodes of the SLI network.

We examined a heterogeneous graph of protein–protein-associations (PPAs) derived from the STRING resource (Szklarczyk *et al.* 2021) together with 2445 synthetic lethal interactions derived from the literature (SLDB; Methods). The classification task in the heterogeneous SLDB graph was to predict novel synthetic lethal interactions. The SLDB graph displayed a skewed node distribution. For instance, the mean degree of the top 20 nodes was 105.9, compared to a mean degree in the entire graph of 2.8. About 2081 of the 2445 edges (85.1%) in the largest component of the SLDB graph involved at least one of the top 20 nodes (Fig. 2 and Supplementary Figs S1 and S2).

### Two methods for sampling negative examples from graphs: UNS and DANS

The evaluation phase requires negative sampling to measure the generalization performance of the edge-prediction model.

**Figure 3.**
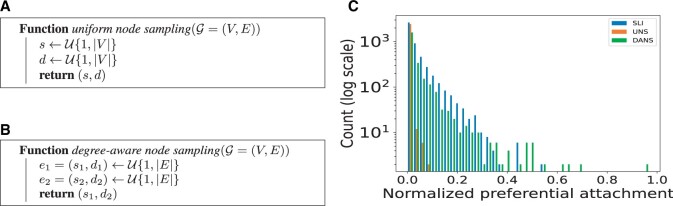
Pseudocode for edge sampling strategies. Both strategies sample two nodes and return the edge that connects the two nodes, but the procedure used for sampling the nodes differs. (A) Uniform node sampling (UNS). The standard method for sampling samples two nodes uniformly to create a “random” edge for negative examples. (B) Degree-aware node sampling (DANS). The method presented here instead samples two edges uniformly to create a random edge from the source node of the first edge and the destination node of the second edge. (C) Preferential attachment (PA). The plot shows PA for positive examples from the SLI graph and edges sampling using the UNS and DANS approaches. See text for details. A Jupyter notebook that performs this analysis and generates panel (C) of this Figure is available on the project GitHub repository.

A common approach for obtaining negative examples samples the source and destination nodes from a uniform distribution that randomly chooses an integer between 1 and |V| corresponding to the nodes of the graph (Fig. 3A). We reasoned that this sampling strategy, which we term *edge sampling by uniform node sampling* (UNS) will produce negative examples whose node degree approximates the degree distribution of the entire KG but may differ from the node degree distribution of the positive examples in many relevant biomedical KGs, because typical biomedical KGs are generally characterized by a non-uniform node-degree distribution (Lima-Mendez and van Helden 2009).

We therefore developed a different sampling mechanism that assigns a number of negative edges to each node proportional to its node degree. We term this method *edge sampling by degree-aware node sampling* (DANS). In this approach, we randomly sample two edges $e_{1}=(s_{1},d_{1}),e_{2}=(s_{2},d_{2})$ from U{1,|E|}, and build a new negative edge by connecting the source node of $e_{1}$ and the destination node of $e_{2}$ (Fig. 3B).

In real-world graphs, there is a minimal likelihood of collisions between existent and non-existent edges using either the UNS or the DANS sampling strategy that can be trivially addressed by repeating the sampling.

To illustrate the effect of the two sampling strategies, we plot the distribution of the product of the node degrees of the two nodes forming edges chosen by the UNS and DANS. In this context, the product of node degrees has been referred to as “preferential node attachment (PA)” (Zhou *et al.* 2009); i.e. if the node degree of node *u* is d(u), then the preferential attachment of edge (u,v) is calculated as PA=d(u)×d(v). We sampled 100 times the number of positive edges in the SLI graph (100×2445) and plotted the distribution of PA for the UNS and DANS sampling approaches as well as for the original (positive) edges. It can be seen that the distribution of edges follow a strikingly different distribution compared to UNS or DANS, whereby the DANS distribution more closely resembles the distribution of the positive edges (Fig. 3C). Indeed, DANS generates negative edges by randomly extracting edges according to a uniform distribution. In this way, source and destination nodes of the negative edges tend to have degrees similar to that of the nodes involved in positive edges, thus resulting in comparable PA distributions between positive SLI edges and negative edges randomly drawn according to DANS.

To investigate the influence of UNS and DANS sampling on a simple classification task, we trained a perceptron (a single layer neural network) to classify SLI interactions using only features derived from node degrees, without taking any other graph features into account. For each edge, we formed a two-dimension integer vector with the degree of each of the nodes that made up the edge. We then compared the results of classification whereby we used UNS and DANS both for the selection of negative edges to train the perceptron (the second phase) with UNS or DANS sampling for the evaluation (third) phase. An equal number of positive and negative examples was chosen. In each experiment, we performed ten-fold cross validation with training size of 0.75.

**Figure 4.**
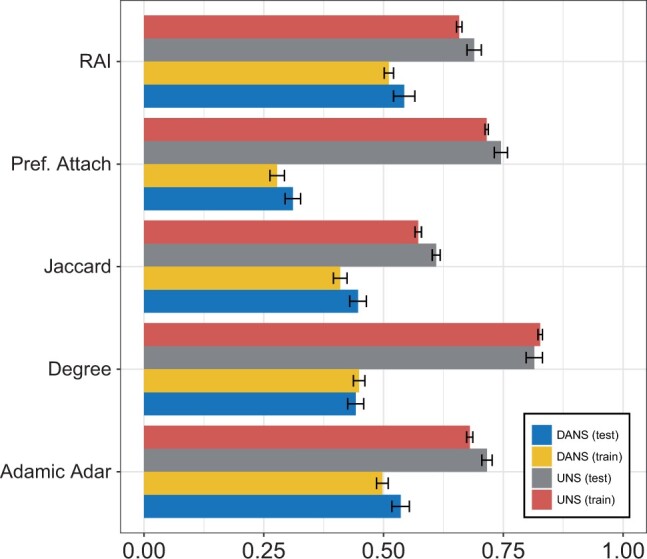
Effects of UNS and DANS sampling on measured classification performance using two-dimensional degree-based features. A perceptron model was trained to predict novel SLI edges. DANS or UNS sampling was used in the evaluation phase. RAI, resource allocation index; Pref. Attach, preferential attachment; Jaccard, Jaccard coefficient. The X-axis shows the Matthews Correlation Coefficient (MCC).

We present results of classification in terms of the Matthews correlation coefficient (MCC), which ranges from −1 for perfect misclassification to +1 for perfect classification, while MCC = 0 is the expected value for a random classifier (Chicco and Jurman 2020). Additional results are presented in Supplementary Figs S2–S5 for four other edge feature generation methods: Adamic-Adar index, Jaccard coefficient, Resource Allocation Index, and Preferential Attachment (Adamic and Adar 2003, Zhou *et al.* 2009) (Fig. 4).

The results of this analysis demonstrate that node-degree bias operates in at least two phases in graph machine learning: in the phase in which the classifier is trained, and also in the phase in which the results of classification are evaluated. The classification models were created identically with the sole exception of the method for choosing negative examples, and yet the measured classification performance differs substantially. To our knowledge, our analysis is the first to show the effects of different sampling strategies on the evaluation phase. If the user of the model is interested in new examples drawn from the same distribution as the known positive examples, then our experiments suggest that at least part of the signal obtained by the classifier using UNS sampling is spurious.

### The influence of negative sampling strategies on GRL edge prediction

We then asked if a similar effect pertains to random walk-based GRL edge prediction. We applied seven different embedding approaches followed by perceptron-based classification of edges in the SLDB graph (Methods).

**Figure 5.**
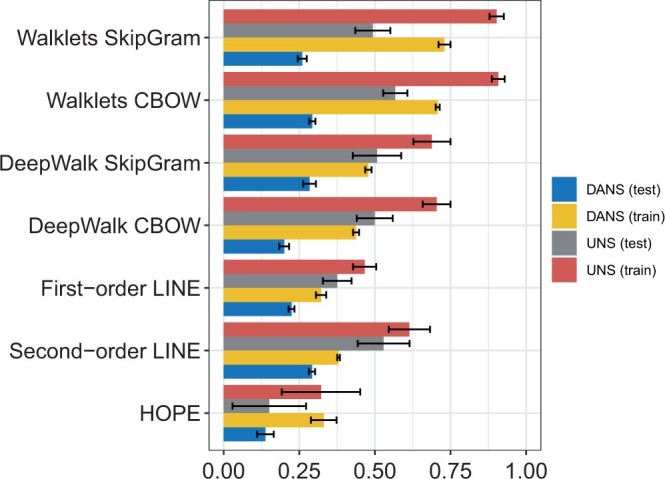
Matthews correlation coefficient for the seven methods applied to edge prediction in the heterogeneous SLDBgraph. The bars show mean ± standard deviation. The X-axis shows the MCC.

We tested the classification performance for the prediction of synthetic lethal interaction edges. The measured classification performance was consistently higher for UNS sampling than for DANS sampling. For instance, Walklets SkipGram displayed an MCC of 0.49 for UNS sampling but only 0.26 for DANS sampling (Fig. 5; see also Supplementary Table S2 for AUROC, AUPRC, and F1 score analysis). For each comparison, the only difference was in the way the negative examples were selected.

The difference in the influence of negative sampling strategies on the SLDB graph is related to the different node distributions or other differences in graph structure. Indeed, the SLDB graph shows a degree distribution that is approximately scale-free, with few nodes of very high degree and many low-degree nodes (Supplementary Figs S1 and S2).

**Figure 6.**
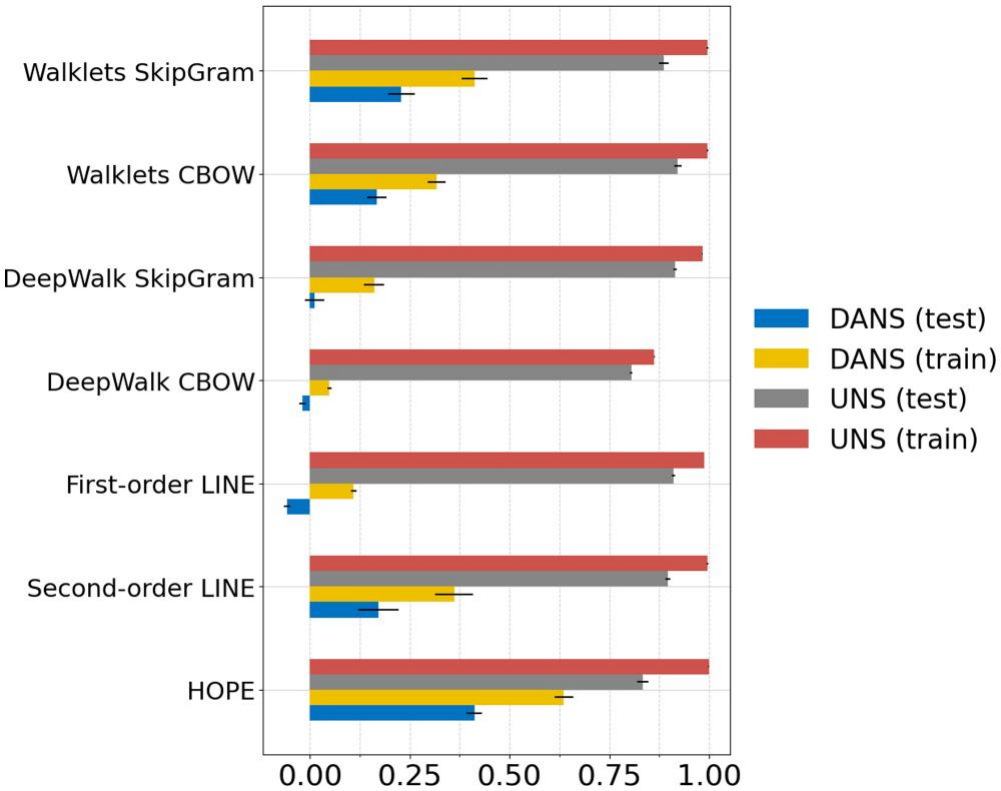
Matthews correlation coefficient for the methods applied to edge prediction in the heterogeneous KG-IDG graph. The bars show mean ± standard deviation. The X-axis shows the MCC.

To confirm this result, we repeated this experiment on KG-IDG, a KG that integrates data related to drug repurposing (Caufield *et al.* 2023a). As before, we produced node embeddings using seven different approaches, and trained a perceptron-based edge prediction model. We then measured the performance of this model in predicting drug to protein edges from this graph. As with SLDB, the classification performance was higher for UNS compared to DANS. For example, the MCC of Walklets SkipGram was 0.90 using UNS sampling but only 0.17 using DANS sampling (Fig. 6). The results from the other five node embeddings strategies were similar: in each case, the MCC using DANS sampling was lower than when using UNS sampling.

## Discussion

Large graphs, including many graphs of interest for biomedical research, commonly follow an approximately scale-free power-law distribution, meaning that the probability P(k) that a node in the network interacts with *k* other vertices decays as a power-law, following $P(k)∼k^{-\gamma }$. This function indicates a high diversity of node degrees, with the lack of a typical degree in the graph motivating the characterization of these graphs as scale-free (Barabasi and Albert 1999, Albert 2005). Intuitively, scale-free networks contain a few hubs that are connected to many other nodes and many nodes with one or only a few connections. It has been known since 2011 that node degree can skew machine-learning predictions in biological graphs (Gillis and Pavlidis 2011). In this work, we characterize the specific influence of negative sampling in generating such biases.

Constructing generalizable graph models of biomedical domains is challenging because most systems of scientific interest have multiple different classes of nodes and relations, and in many cases, our knowledge is so incomplete that comprehensive gold-standard data sets for training ML classifiers do not exist. An ML algorithm is said to be biased if its results are systematically wrong due to incorrect assumptions of the ML process. Biases can inflate the measured prediction performance of algorithms (Eid *et al.* 2021). In this work we have explored the relationship between sampling of negative examples and measured performance of shallow graph representation learners. Our results demonstrate that if the positive and negative samples have a different node degree distribution, then strategies for sampling negative examples for the evaluation that do not take node degree into account can greatly affect the estimate performance of classification algorithms.

While the classification performance of a given ML model depends on several crucial factors, including the amount of information in the training dataset, the training algorithm and its parameter settings, it is important to realize that the validity and reliability of the model assessment strongly depends on the distribution of the novel data the model is meant to classify. We note that the effect we have described here is not the same as overfitting. Rather, what we have shown is essentially that standard (UNS-based) approaches to evaluating classifiers in biomedical KGs risk comparing apples and oranges because the distribution of the positive examples in model training and evaluation is different. While the results that are estimated using UNS seem to be accurate, they are derived from an artificially “easy” classification task that reflects differences in node degree between positive edges (typically high degree) and uniformly chosen edges (typically low degree). We argue that in many cases this does not reflect the actual biomedical problem, which is to identify novel edges of high degree nodes in an existing KG. We have shown that the DANS approach provides a more relevant estimation of performance of ML classifiers in this situation. For instance, if a biologist wants to find new SLI interactions that involve one of the genes shown in Fig. 2C, the node degree of such candidates will be more similar to the node degree sampled by DANS than that from UNS.

Our review of other software packages for RW-GRL revealed that the uniform node sampling procedure is used during training by the gensim package (Řehůřek and Sojka 2010) that is widely used as to develop algorithms to perform embedding such as node2vec (Grover and Leskovec 2016). The methods used for sampling negative examples are rarely described in detail in the methods of many publications on the topic. Our results suggest that users should take this into account and report on the approach to negative sampling.

Limitations of our analysis include the restriction to random-walk-based GRL. We have not performed exhaustive parameter optimization; however, the parameters used in the results presented here are typical for the algorithms, and the effects described in this work do not pertain to the model training phase but instead to evaluation. It might be the case that some models, datasets, or parameter combinations obtain similar estimates of prediction accuracies when UNS and DANS are used. For instance, we observed no substantial difference in UNS and DANS inflated results, when the HOPE model is used for prediction of SLIs. However, we observe substantial differences for the vast majority of models tested. Therefore, we recommend that practitioners of random-walk based graph representation learning always investigate performance of their models using both UNS and DANS sampling to characterize potential differences. Further work will delve into the exploration of this issue for specific models and prediction tasks.

## Conclusions

Negative samples should be meaningful for the classification task at hand, and inappropriate sampling mechanisms may lead to biased evaluation. Negative samples that differ substantially from the positive samples in one or more characteristics can lead to over-optimistic evaluations. Conversely, negative samples that are too similar to (or even collide with) the positive samples may lead to overly poor evaluations.

We recommend that practitioners of edge prediction in biological networks carefully evaluate the node degree distribution of samples chosen for the negative and positive examples. Results of training with the standard uniform node selection schema and the node-degree selection approach presented here should be compared.

## Supplementary Material

vbae036_Supplementary_Data

## Data Availability

No primary data was generated for this work. The graph data investigated here can be imported using the functions demonstrated in the illustrative Jupyter notebooks at https://github.com/monarch-initiative/negativeExampleSelection.

## Code availability

The scripts used to generate the results described in this manuscript are available together with several illustrative Jupyter notebooks at https://github.com/monarch-initiative/negativeExampleSelection under the GNU General Public License version 3.
