# Supplementary material for: Node-degree aware edge sampling mitigates inflated classification performance in biomedical random walk-based graph representation learning
Source: Bioinform Adv. 2024 Mar 4;4(1):vbae036. doi: 10.1093/bioadv/vbae036 (PMC10994718; doi:10.1093/bioadv/vbae036)
Supplement: vbae036_Supplementary_Data [file vbae036_supplementary_data.pdf]

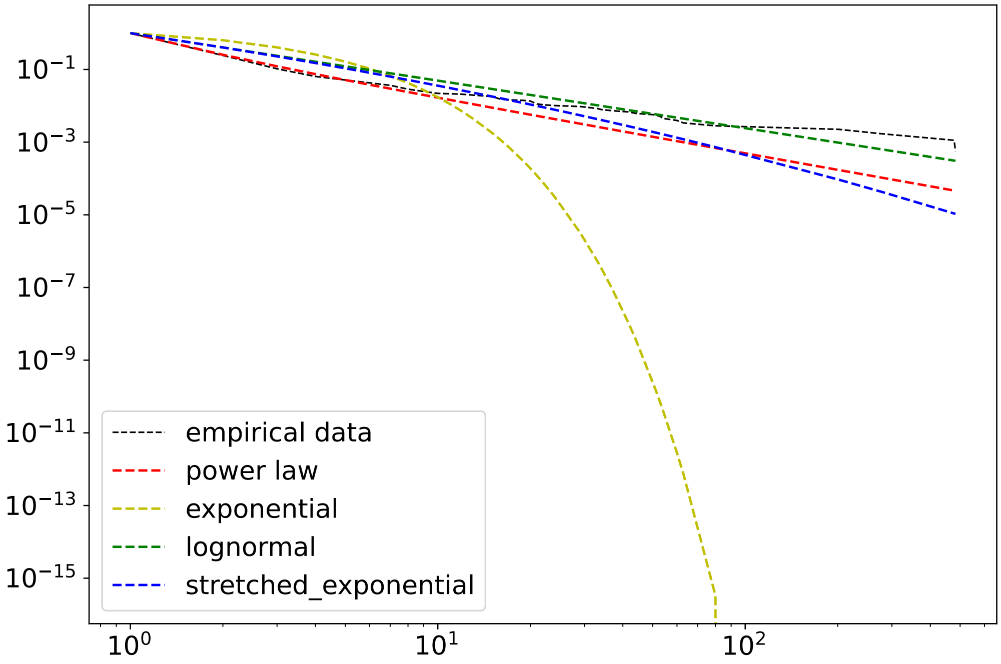

Figure S1: Analysis of the degree distribution of the SLDB graph. The observed distribution was consistent with a power-law distribution with $\alpha = 2.5$. No other tested distribution provided a significantly better fit. The largest connected component of the graph was analyzed with the the powerlaw Python package [1].

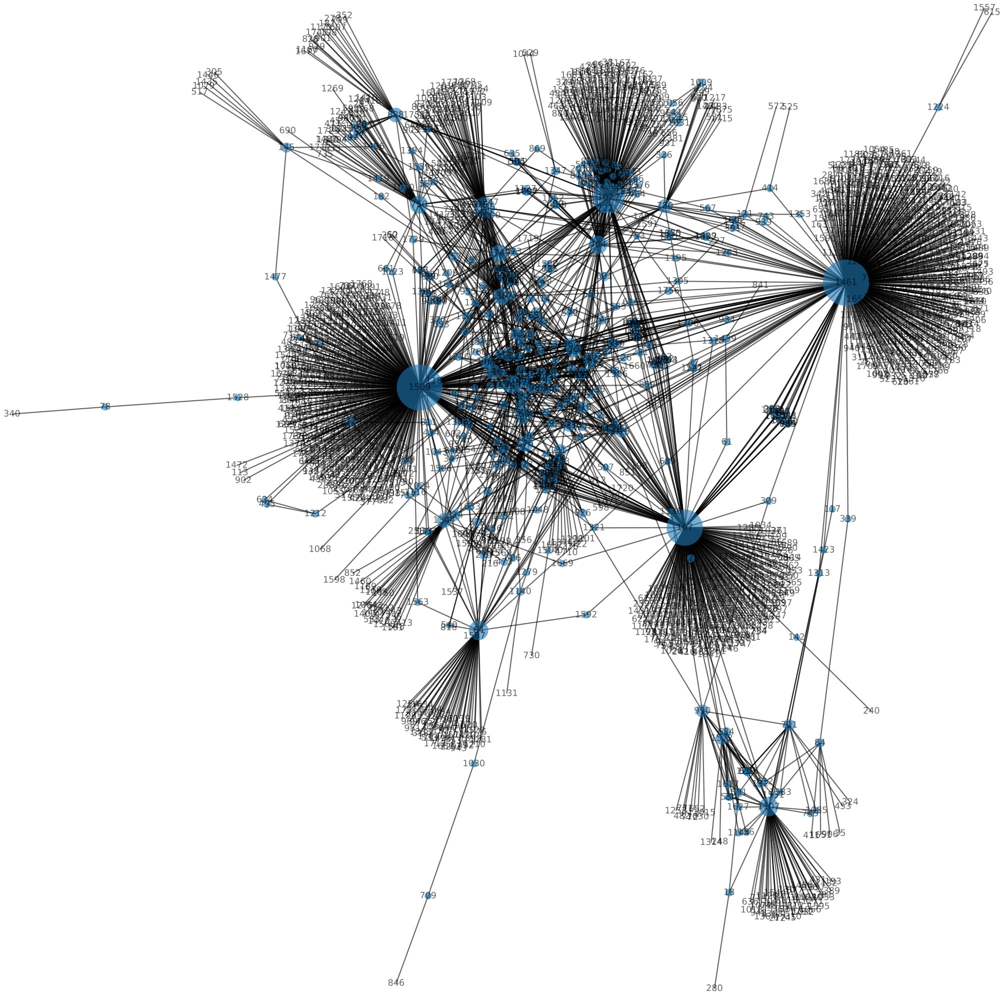

Figure S2: Visualization of the SLDB network. Only the largest connected component is shown. Nodes with degree of 2 or more are shown in blue. Inspection of the SLDB graph showed it to have a highly skewed degree distribution with a few hubs, i.e., nodes that are highly connected to other nodes in the network, and many nodes with few connections. See Supplemental Figure **S2** for an analysis of the degree distribution of this graph.

Characteristic	SLDB	STRING	SLDB/STRING
connected components	6	98	1
size of smallest component	2	2	16715
size of largest component	1765	16582	16715
nodes	1775	16812	16715
edges	2445	252953	255253
diameter	9.0	14.0	14.0
clustering coefficient	58.4	5617.6	5588.4
density	0.0016	0.0018	0.0017
mean node degree	2.8	30.5	30.5
median node degree	1	13	13
transitivity	0.0034	0.31	0.30

Table S1: Network characteristics of the SLDB, STRING, and SLDB/STRING (composite) graphs. Network characteristics were calculated with the GRAPE library [**?**]. The composite SLDB/STRING graph was used for the analysis and is refered to simply as "SLDB" in the main manuscript.

Model	Evaluation	F1	AUROC	AUPRC	MCC
DeepWalk CBOW	DANS (test)	0.815 ± 0.020	0.889 ± 0.012	0.876 ± 0.018	0.647 ± 0.033
—"—	UNS (test)	0.843 ± 0.025	0.922 ± 0.020	0.919 ± 0.012	0.716 ± 0.045
—"—	DANS (train)	0.876 ± 0.036	0.934 ± 0.022	0.913 ± 0.013	0.751 ± 0.069
—"—	UNS (train)	0.904 ± 0.042	0.956 ± 0.025	0.945 ± 0.012	0.814 ± 0.078
DeepWalk SkipGram	DANS (test)	0.862 ± 0.025	0.912 ± 0.013	0.869 ± 0.022	0.736 ± 0.031
—"—	UNS (test)	0.888 ± 0.043	0.945 ± 0.033	0.933 ± 0.040	0.796 ± 0.072
—"—	DANS (train)	0.918 ± 0.018	0.948 ± 0.014	0.900 ± 0.019	0.837 ± 0.031
—"—	UNS (train)	0.945 ± 0.036	0.972 ± 0.024	0.952 ± 0.033	0.892 ± 0.068
First-order LINE	DANS (test)	0.874 ± 0.014	0.942 ± 0.011	0.946 ± 0.011	0.747 ± 0.038
—"—	UNS (test)	0.912 ± 0.010	0.961 ± 0.006	0.968 ± 0.005	0.831 ± 0.017
—"—	DANS (train)	0.901 ± 0.018	0.964 ± 0.009	0.962 ± 0.010	0.797 ± 0.041
—"—	UNS (train)	0.938 ± 0.006	0.981 ± 0.004	0.982 ± 0.003	0.878 ± 0.012
HOPE	DANS (test)	0.797 ± 0.023	0.898 ± 0.007	0.907 ± 0.008	0.651 ± 0.011
—"—	UNS (test)	0.831 ± 0.039	0.933 ± 0.013	0.954 ± 0.006	0.738 ± 0.043
—"—	DANS (train)	0.805 ± 0.020	0.913 ± 0.004	0.916 ± 0.010	0.661 ± 0.010
—"—	UNS (train)	0.839 ± 0.036	0.958 ± 0.007	0.966 ± 0.003	0.747 ± 0.041
Second-order LINE	DANS (test)	0.865 ± 0.019	0.935 ± 0.015	0.941 ± 0.014	0.728 ± 0.051
—"—	UNS (test)	0.910 ± 0.010	0.960 ± 0.006	0.968 ± 0.004	0.832 ± 0.015
—"—	DANS (train)	0.890 ± 0.031	0.957 ± 0.017	0.956 ± 0.017	0.774 ± 0.068
—"—	UNS (train)	0.936 ± 0.002	0.981 ± 0.002	0.983 ± 0.001	0.874 ± 0.004
Walklets CBOW	DANS (test)	0.866 ± 0.032	0.935 ± 0.017	0.911 ± 0.024	0.768 ± 0.038
—"—	UNS (test)	0.880 ± 0.036	0.973 ± 0.005	0.968 ± 0.008	0.802 ± 0.046
—"—	DANS (train)	0.965 ± 0.015	0.989 ± 0.006	0.972 ± 0.016	0.931 ± 0.027
—"—	UNS (train)	0.979 ± 0.019	0.996 ± 0.002	0.988 ± 0.006	0.960 ± 0.034
Walklets SkipGram	DANS (test)	0.775 ± 0.066	0.904 ± 0.015	0.885 ± 0.022	0.659 ± 0.063
—"—	UNS (test)	0.791 ± 0.067	0.950 ± 0.035	0.958 ± 0.020	0.699 ± 0.070
—"—	DANS (train)	0.968 ± 0.013	0.985 ± 0.009	0.972 ± 0.018	0.938 ± 0.022
—"—	UNS (train)	0.984 ± 0.015	0.994 ± 0.005	0.991 ± 0.004	0.969 ± 0.027

Table S2: SLDB/STRING: Random-walk graph representation learning bias analysis. F1: F1 score (harmonic mean of the precision and recall); AUROC: area under the receiver operating characteristics; AUPRC: area under the precision recall curve. Results are shown for train and test phases using the two different negative example selection strategies investigated in this work: uniform node sampling, and node-degree sampling.

References

[1] J. Alstott, E. Bullmore, and D. Plenz. Powerlaw: a Python package for analysis of heavy-tailed distributions. *PLoS One*, 9(1):e85777, 2014.

[2] Luca Cappelletti, Tommaso Fontana, Elena Casiraghi, Vida Ravanmehr, Tiffany J. Callahan, Marcin P. Joachimiak, Christopher J. Mungall, Peter N. Robinson, Justin T. Reese, and Giorgio Valentini. Grape: fast and scalable graph processing and embedding. *CoRR*, abs/2110.06196, 2021.
